# AliSim-HPC: parallel sequence simulator for phylogenetics

**DOI:** 10.1093/bioinformatics/btad540

**Published:** 2023-09-01

**Authors:** Nhan Ly-Trong, Giuseppe M J Barca, Bui Quang Minh

**Affiliations:** School of Computing, College of Engineering, Computing and Cybernetics, Australian National University, Canberra, ACT 2600, Australia; School of Computing, College of Engineering, Computing and Cybernetics, Australian National University, Canberra, ACT 2600, Australia; School of Computing, College of Engineering, Computing and Cybernetics, Australian National University, Canberra, ACT 2600, Australia

## Abstract

**Motivation:**

Sequence simulation plays a vital role in phylogenetics with many applications, such as evaluating phylogenetic methods, testing hypotheses, and generating training data for machine-learning applications. We recently introduced a new simulator for multiple sequence alignments called AliSim, which outperformed existing tools. However, with the increasing demands of simulating large data sets, AliSim is still slow due to its sequential implementation; for example, to simulate millions of sequence alignments, AliSim took several days or weeks. Parallelization has been used for many phylogenetic inference methods but not yet for sequence simulation.

**Results:**

This paper introduces AliSim-HPC, which, for the first time, employs high-performance computing for phylogenetic simulations. AliSim-HPC parallelizes the simulation process at both multi-core and multi-CPU levels using the OpenMP and message passing interface (MPI) libraries, respectively. AliSim-HPC is highly efficient and scalable, which reduces the runtime to simulate 100 large gap-free alignments (30 000 sequences of one million sites) from over one day to 11 min using 256 CPU cores from a cluster with six computing nodes, a 153-fold speedup. While the OpenMP version can only simulate gap-free alignments, the MPI version supports insertion–deletion models like the sequential AliSim.

**Availability and implementation:**

AliSim-HPC is open-source and available as part of the new IQ-TREE version v2.2.3 at https://github.com/iqtree/iqtree2/releases with a user manual at http://www.iqtree.org/doc/AliSim.

## 1 Introduction

**Figure 1.**
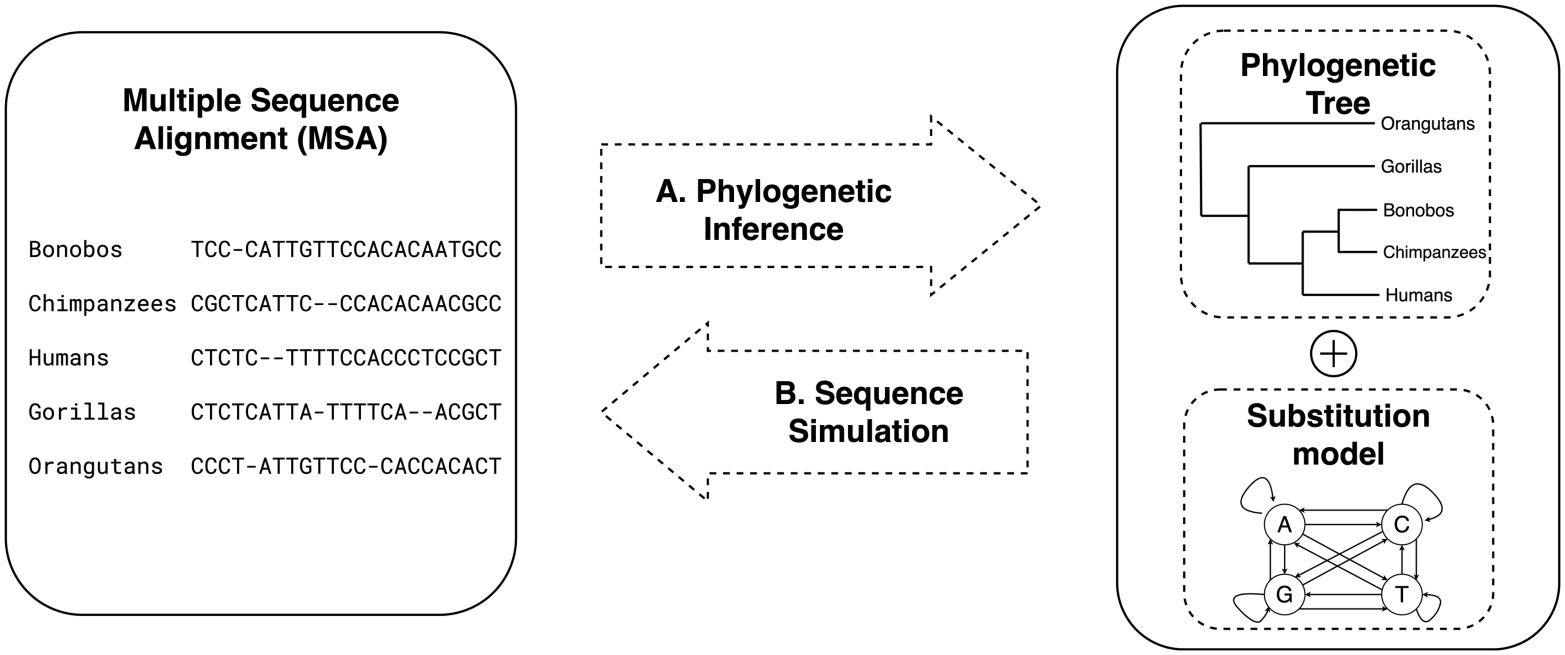
Phylogenetic inference (A) to infer a phylogenetic tree and a substitution model from an input multiple sequence alignment; and Sequence simulation (B) to generate multiple sequence alignment(s) from a phylogenetic tree and a substitution model.

Phylogenetic inference is an important problem in bioinformatics, which aims to reconstruct a phylogenetic tree that describes the evolutionary relationship among a set of organisms (Felsenstein 2004, Lemey *et al.* 2009). Typical phylogenetic inference methods require a multiple sequence alignment (MSA) containing DNA or amino-acid sequences as input and return a phylogenetic tree and a substitution model as output (Fig. 1A). In a phylogenetic tree, tips (leaves) represent the organisms in the MSA, internal nodes denote the extinct common ancestors. A substitution model is typically a Markov process that describes the rates of changes between nucleotides (for DNA sequences) or amino acids (for protein sequences).

Sequence simulation is an inverse problem of phylogenetic inference: we want to simulate MSAs from a phylogenetic tree and a substitution model (Fig. 1B). Simulated data has many applications in phylogenetics, such as evaluating phylogenetic methods (Garland *et al.* 1993, Kuhner and Felsenstein 1994, Tateno *et al.* 1994, Huelsenbeck 1995), testing hypothesis (Goldman 1993, Adell and Dopazo 1994, Schöniger and von Haeseler 1999), and more recently, generating data for training new machine-learning methods (Abadi *et al.* 2020, Leuchtenberger *et al.* 2020, Ling *et al.* 2020, Suvorov *et al.* 2020).

We recently introduced a new sequence simulator, AliSim (Ly-Trong *et al.* 2022), which outperformed existing simulators [i.e. Seq-Gen (Rambaut and Grassly 1997), Dawg (Cartwright 2005), INDELible (Fletcher and Yang 2009), and phastSim (De Maio *et al.* 2022)] regarding both running time and memory footprint. Since its publication in 2022, AliSim has already been used in several studies using machine learning (Legall and Salvador 2022, Smith and Hahn 2022, Suvorov and Schrider 2022).

Many applications require simulations of a vast number of MSAs. To do so, users can manually parallelize it by independently launching several AliSim jobs in a high-end server or a cluster (e.g. with sbatch), each job simulates a subset of MSAs. However, the RAM requirement of this manual approach grows linearly with the number of jobs, which may limit the number of jobs that can run in parallel. Moreover, this approach does not work well in a particular scenario of simulating an extremely large MSA due to the sequential implementation of AliSim and all other simulation software mentioned above. For example, AliSim takes hours to simulate an MSA with millions of sequences or sites (Ly-Trong *et al.* 2022). Our main aim here is to solve both problems, i.e. not only to speed up the simulation of single large MSAs but also to reduce the memory footprint. Parallelization has been widely used for phylogenetic inference methods (Altekar *et al.* 2004, Bouckaert *et al.* 2014, Kozlov *et al.* 2015, 2019, Morel *et al.* 2019, Minh *et al.* 2020) but has not yet been employed in simulation software.

In this paper, we introduce AliSim-HPC, a high-performance computing version of AliSim. AliSim-HPC parallelizes the simulations at both multi-core and multi-CPU levels using OpenMP (Chapman *et al.* 2007) and the message passing interface (MPI) (Gropp *et al.* 1998), respectively. We first present two multi-threading algorithms to parallelize the simulation of a single (large) gap-free alignment with the OpenMP library. Next, we utilize the MPI library to parallelize the simulation of many alignments across distributed CPUs. We can thus deploy AliSim-HPC that combines OpenMP and MPI on a high-performance computing cluster with many nodes. We note that the proposed algorithms are generally applicable to shared and distributed-memory paradigms. We only chose OpenMP and MPI because these two libraries have already been used in IQ-TREE. While the OpenMP algorithms can only simulate gap-free alignments, the MPI version fully supports insertion–deletion models (Indels) like the original AliSim.

AliSim-HPC shows an excellent scaling behavior: it reduces the simulation time of 100 large alignments (30 000 sequences of one million sites) without gaps from over one day to 11 min by using 256 CPU cores (153-fold speedup). AliSim-HPC is flexible: it can run on a personal computer with multi-threading, as well as on a distributed-memory cluster with many CPUs and multiple cores per CPU.

Our contributions are 4-fold. First, this is the very first-time high-performance computing techniques are applied to phylogenetic sequence simulators. Second, we provide AliSim-HPC as an extension of IQ-TREE (Nguyen *et al.* 2015, Minh *et al.* 2020), an open-source and widely used phylogenetic software, thus maximizing its usage and benefit to the user community. Third, we demonstrate that AliSim-HPC can efficiently simulate large genomic data sets, thus facilitating large-scale benchmarking of phylogenetic methods and providing training data for machine learning-based applications. And fourth, we provide practical recommendations on the choice of the number of threads per process and multi-threading algorithms for simulating large genomic data sets.

## 2 Materials and methods

### 2.1 The sequential AliSim algorithm

**Figure 2.**
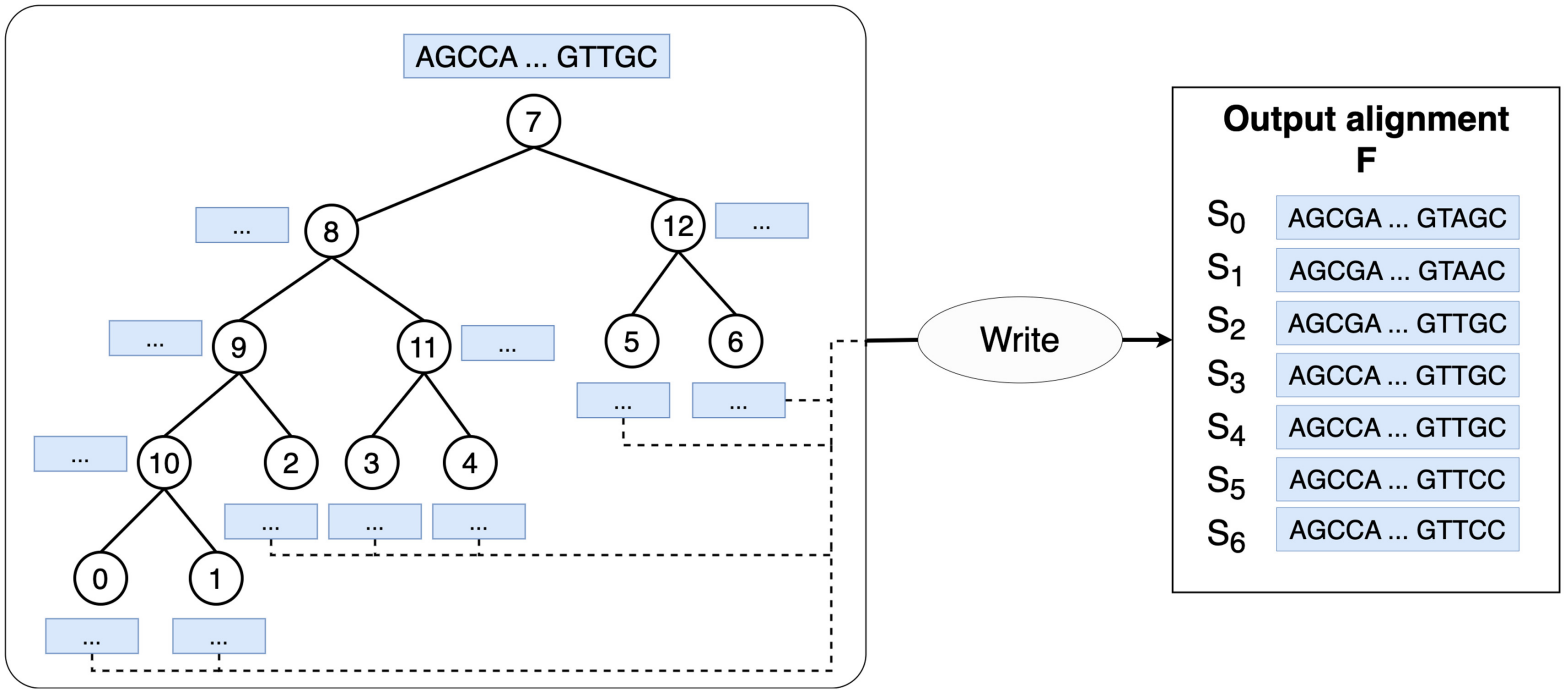
Illustration of the sequential AliSim algorithm to simulate a single alignment along an example tree. It starts by generating a sequence $S_{7}$ at the root node 7, and subsequently, performs a preorder traversal visiting nodes 8, 9, 10, 0, 1, 2, 11, 3, 4, 12, 5, and 6. AliSim simulates $S_{8}$ based on $S_{7}$ and the substitution model *M*, and so on. At tip nodes 0 to 6, it writes $S_{0}$ to $S_{6}$ to an output alignment.

Here, we provide a brief summary of the published AliSim algorithm (Ly-Trong *et al.* 2022). Assuming that we want to simulate an alignment with *N* sequences, each of which contains *L* sites from a phylogenetic tree *T* and a substitution model *M*. Let $S_{j}$ denote the sequence at node *j* of tree *T*. AliSim first generates a sequence with *L* sites at the root of the tree based on the state frequencies of model *M* (Fig. 2). Then, AliSim traverses tree *T* in a preorder manner to simulate a new sequence at each node based on the sequence of its parent node and the substitution model *M* (Ly-Trong *et al.* 2022). At tips, AliSim writes the simulated sequences to an MSA file. To generate many alignments, AliSim repeats this process sequentially. We present the sequential AliSim in Algorithm 1.

Algorithm 1. **SequentialAliSim(*T*, *M*, *L*, *F*)**

**Input:** a phylogenetic tree *T* with *N* tips; a substitution model *M*; a sequence length *L*.

**Output:** an alignment file *F* containing *N* sequences of length *L*.

1. Generate a sequence $S_{\mathrm{root}}$ of length *L* at the root of tree *T* based on model *M*.

2. Perform a preorder traversal of tree *T*, at each node *j*, do:

  2.1. Simulate a new sequence $S_{j}$ based on the sequence at the parent of node *j* and model *M*.

  2.2. If *j* is a tip, write $S_{j}$ to file *F*.

Thanks to a memory-saving technique (Ly-Trong *et al.* 2022), the memory complexity of the sequential AliSim algorithm is O(N)+O(D*L), where *D* is the depth of tree *T*. The first O(N) and the second O(D*L) terms represent the memory to store the tree structure and the simulated sequences, respectively.

### 2.2 AliSim-OpenMP

**Figure 3.**
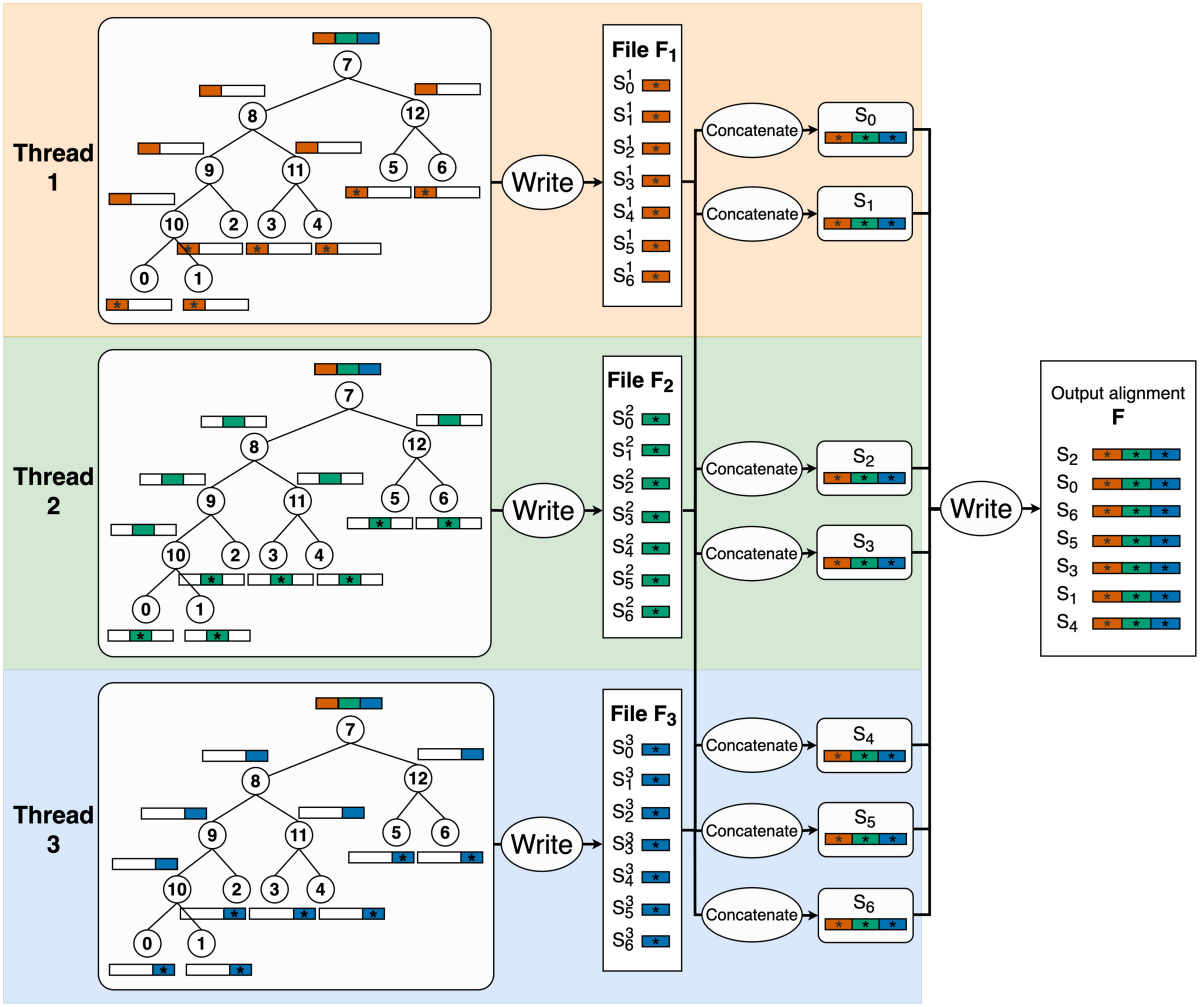
Illustration of the AliSim-OpenMP algorithm using external memory to simulate an alignment without gaps with three threads. These threads execute the sequential AliSim algorithm independently to generate three temporary files $F_{1},F_{2},F_{3}$; each file contains seven subsequences of length $\frac{L}{3}$. Then, thread 1 reconstructs two sequences $S_{0}$ and $S_{1}$ by concatenating their subsequences from all three temporary files. At the same time, thread 2 reconstructs $S_{2}$ and $S_{3}$ while thread 3 reconstructs $S_{4},S_{5}$, and $S_{6}$. The concatenated sequences are written one by one to the output alignment.

We now introduce two different algorithms to parallelize AliSim using OpenMP, which assumes no insertion–deletions (Indels) as follows. Without Indels, the evolution of sites in the MSA is independent, which naturally allows a parallel scheme for simulating an alignment with OpenMP (Fig. 3): each thread independently simulates a continuous block of the MSA with a roughly similar length. Given *K* threads, each thread *i* simply executes SequentialAliSim($T,M,\frac{L}{K},F_{i}$) to generate a temporary file $F_{i}$ containing *N* sequences of length $\frac{L}{K}$.

Next, we need to combine individual $F_{i}$ files into a single alignment file *F*. AliSim invokes another parallel section, where each thread reads a subset of roughly $\frac{N}{K}$ sequences across all temporary files, concatenates the subsequences into the full sequences of length *L*, then writes the full concatenated sequences to file *F* in a critical OpenMP section because file writing operations are not thread-safe. This algorithm is called “AliSim-OpenMP using external memory” because it creates temporary files to store intermediate alignments and is outlined in Algorithm 2.

Algorithm 2 has the same memory complexity of O(N)+O(D*L) as the sequential AliSim algorithm, where *D* is the depth of tree *T*. However, it consumes double the amount of external memory to store temporary files.

Algorithm 2 contains two parallel sections. The first section is embarrassingly parallel without any inter-thread communications; thus, we expect this section to gain linear speedup. However, the second section can be the main bottleneck due to too many I/O operations. A quick solution to deal with this problem would be to re-implement $F_{i}$ as an internal memory storage. However, it requires an additional memory of O(N*L), which by large exceeds O(D*L) and is therefore undesirable for large alignment simulations.

Algorithm 2. **AliSimOpenMP_EM(*T*, *M*, *L*, *F*, *K*)**

**Input:** a phylogenetic tree *T* with *N* tips; a substitution model *M*; a sequence length *L*; a number of threads *K*.

**Output:** a gap-free alignment file *F* containing *N* sequences of length *L*.

1. For each thread *i* from 1 to *K*:

 Call SequentialAliSim($T,M,\frac{L}{K},F_{i}$) to generate a temporary alignment file $F_{i}$

2. For each thread *i* from 1 to *K*:

 For each tip index *j* from $(i-1)*\frac{N}{K}+1$ to $i*\frac{N}{K}$:

  2.1. Read subsequences $S_{j}^{i}$ from all temporary files $F_{i}$, where i∈{1,2,…,K}.

  2.2. Concatenate these subsequences $S_{j}^{i}$ into the full-length sequence $S_{j}$.

  2.3. Write $S_{j}$ to file *F* in a critical OpenMP section.

**Figure 4.**
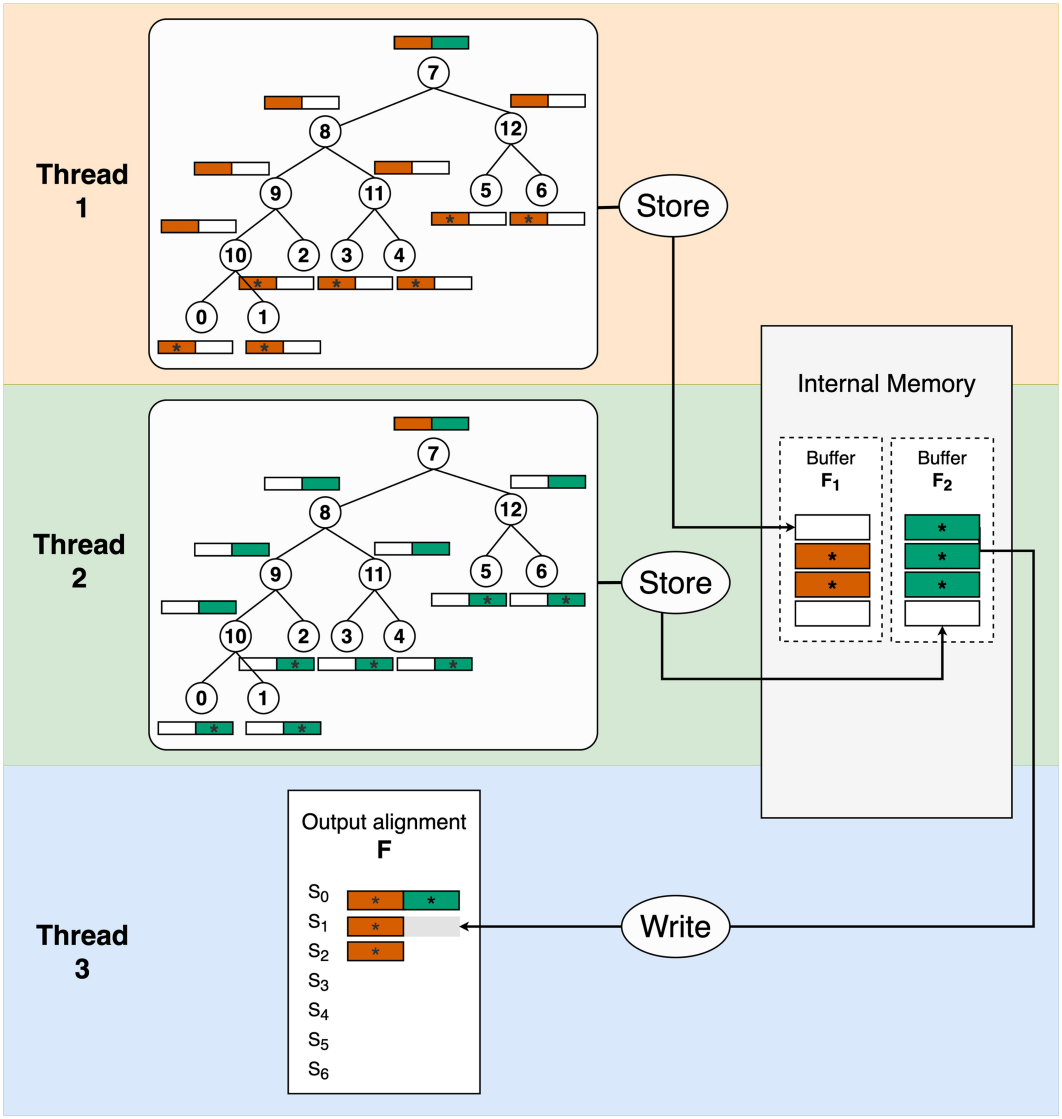
Illustration of the AliSim-OpenMP algorithm using internal memory to simulate an alignment without gaps with three threads. Threads 1 and 2 execute a modified version of the sequential AliSim algorithm independently to simulate subsequences of $S_{0}$ to $S_{6}$ of length $\frac{L}{2}$ and store these subsequences in the corresponding buffers $F_{1}$ and $F_{2}$. Thread 3 continuously accesses buffers $F_{1}$ and $F_{2}$ to write subsequences into their precomputed positions in the output alignment *F* and then free the corresponding memory in the buffers. Thread 3 repeats that process until threads 1 and 2 are finished and all subsequences are outputted to the output alignment.

Therefore, we designed another algorithm called “AliSim-OpenMP using internal memory” (Fig. 4) to avoid writing temporary files as follows. We allocate K−1 threads, each thread simulates one of the (K−1) blocks of the MSA by calling a modified version of SequentialAliSim($T,M,\frac{L}{K-1},F_{i}$), where $F_{i}$ is now redesigned as a buffer in the internal memory. Whereas the last thread *K* is dedicated to only writing the buffers into the output file *F*.

To reduce the RAM consumption, each thread does not store all *N* subsequences in its buffer $F_{i}$, but only a fraction N*λ of subsequences, where λ is a parameter between 0 and 1. Whenever each “simulating” thread *i* (from 1 to K−1) simulated a subsequence $S_{j}^{i}$ at tip *j*, that thread will compute the absolute position of $S_{j}^{i}$ in the output file *F*, then store $S_{j}^{i}$ and its position to $F_{i}$ if $F_{i}$ has some free memory. Otherwise, thread *i* will need to wait until $F_{i}$ becomes available.

The I/O thread *K* continuously monitors the buffers. Whenever there is any subsequence $S_{j}^{i}$ stored in any buffer $F_{i}$, it will jump to the precomputed position of $S_{j}^{i}$ in file *F* and write $S_{j}^{i}$ then free the corresponding memory in $F_{i}$. The I/O thread then checks the next buffer in the round-robin fashion (either $F_{i+1}$ if i<K−1 or $F_{1}$ if i=K−1). This ensures a relative balance in memory availability among the buffers. This algorithm is outlined in Algorithm 3.

Algorithm 3. **AliSimOpenMP_IM(*T*, *M*, *L*, *F*, *K*)**

**Input:** a phylogenetic tree *T* with *N* tips; a substitution model *M*; a sequence length *L*; a number of threads *K*.

**Output:** a gap-free alignment file *F* containing *N* sequences of length *L*.

For each thread *i* from 1 to K−1:

  Call the modified SequentialAliSim($T,M,\frac{L}{K-1},F_{i}$) to simulate subsequences, store those sequences and their absolute positions in the alignment file *F* into buffer $F_{i}$.

For thread *K*:

  1. Initialize i=1.

  2. While any of the previous threads is not finished:

      Find a subsequence $S_{j}^{i}$ in $F_{i}$.

     Jump to the pre-computed position in file *F*, then write $S_{j}^{i}$.

    Free the corresponding memory in $F_{i}$.

    Set i=i mod (K−1)+1.

  3. When all previous threads are finished:

    Write all $F_{i}$ to file *F* for all *i* from 1 to K−1.

The memory complexity of this algorithm is O(N)+O(D*L+N*λ*L). Small λ will increase the waiting time of the “simulating” threads, thus potentially increasing the runtime. Whereas large λ will increase the RAM consumption. To balance the trade-off between runtime and RAM consumption, we set the default λ to $\min(\frac{(K-1)*2}{N},1)$ because with more threads (higher *K*), each thread needs to simulate shorter subsequences, which is faster than having fewer threads, and therefore a larger buffer size is needed.

The two AliSim-OpenMP algorithms introduced above have their own advantages and disadvantages depending on the simulating conditions, but they complement each other.

The design of AliSim-OpenMP is based on the assumption that different sites in the alignment evolve independently. However, this assumption does not hold for some advanced models, such as Indels. In the following, we introduce AliSim-MPI, which can simulate many alignments with Indels.

### 2.3 AliSim-MPI

A practical demand is to simulate many alignments. Naively, users can manually run several AliSim jobs on a cluster, each simulating one alignment. To make it more convenient, we developed AliSim-MPI, which automatically distributes this task on a cluster within a single run. Specifically, to simulate *H* alignments using *P* MPI processes, AliSim-MPI simulates roughly $\left\lceil \frac{H}{P}\right\rceil$ alignments per process. These processes perform simulations independently and write separate alignment files. No communication is needed between the processes. The memory complexity is proportional to the number of processes: O(P*N)+O(P*D*L). Unlike AliSim-OpenMP, this algorithm perfectly supports Indels as the sequential version of AliSim.

### 2.4 AliSim-HPC for high-performance computing systems

We now combine AliSim-OpenMP and AliSim-MPI to enable simulations on a large cluster with *P* processes, each having *K* threads (i.e. a total of P*K threads are run in parallel). Note that AliSim-HPC does not support Indels if K>1. The AliSim-HPC algorithm is outlined in Algorithm 4.

Algorithm 4. **AliSimHPC(*T*, *M*, *L*, *H*, *P*, *K*)**

**Input:** a phylogenetic tree *T* with *N* tips; a substitution model *M*; a sequence length *L*; a number of alignments *H*; a number of processes *P*; a number of threads per process *K*.

**Output:** *H* alignment files $F^{z}$, *where*  z∈{1,2,…,H}; each alignment file contains *N* sequences of length *L*.

For each process *w* from 1 to *P*:

  For *z* from 1 to *H*:

   If z mod P+1=w:

   Call *AliSimOpenMP_EM*($T,M,L,F^{z},K$) or *AliSimOpenMP_IM*($T,M,L,F^{z},K$).

We thereby refer to the two variants of AliSim-HPC that integrate AliSim-OpenMP using external memory and internal memory as AliSim-HPC-EM and AliSim-HPC-IM, respectively.

The memory complexity is O(P*N)+O(P*D*L) for AliSim-HPC-EM; and O(P*N)+O(P*D*L+P*N*λ*L) for AliSim-HPC-IM.

### 2.5 Random generator initialization

Reproducibility is strongly desirable in any software, which involves random number generation. To ensure this, AliSim-HPC employs The Scalable Parallel Random Number Generators Library (SPRNG) (Mascagni and Srinivasan 2000) and allows users to specify a random number generator seed *r*. It then computes a unique seed number for each thread of each process as r+p*1000+k, where *p* and *k* denote the process and thread IDs, respectively. If *r* is not provided, it will be set to the current microsecond of the CPU.

### 2.6 Benchmark setup

We evaluated the performance of the two variants of AliSim-HPC (using external and internal memory) compared with the sequential AliSim on the Gadi supercomputer (https://nci.org.au/our-systems/hpc-systems), a cluster of 3200 nodes with a total of 155 000 CPU cores, 567 TB of RAM, and 640 GPUs. We also benchmarked the manual approach, where users submit several sequential AliSim jobs. We employed up to six computing nodes, each of which has 2 × 24-core Intel Xeon Platinum 8274 (Cascade Lake) 3.2 GHz CPUs and 400 GB SSD. We run all experiments with Open-MPI v4.1.3 (https://www.open-mpi.org) and OpenMP 4.5.

We measured the strong scaling behavior and total RAM consumption when simulating 100 large alignments without Indels. Due to computational resource constraints, we set the maximum number of processes, number of threads per process, and number of jobs at 32. By varying the number of processes *P* and the number of threads per process *K* at 1, 2, 4, 8, 16, and 32, we formed a total of 33 combinations, such that the total number of CPU cores (the number of processes times the number of threads) was up to 256. Similarly, we varied the number of jobs at 1, 2, 4, 8, 16, and 32. We simulated two types of large alignments: 1M sequences of 30K sites, which we called a “deep-alignment”; and 30K sequences of 1M sites, which we called a “long-alignment”. The input phylogenetic trees were randomly drawn under the Yule–Harding model (Yule 1925, Harding 1971) and exponentially distributed branch lengths with a mean of 0.1. For the model of evolutions, we applied the general time reversible (GTR) (Tavaré 1986) with an invariant site proportion of 0.2 and discrete Gamma rate heterogeneity (Gu *et al.* 1995) with a Gamma shape of 0.5.

## 3 Results

### 3.1 Two AliSim-OpenMP algorithms complemented each other

**Figure 5.**
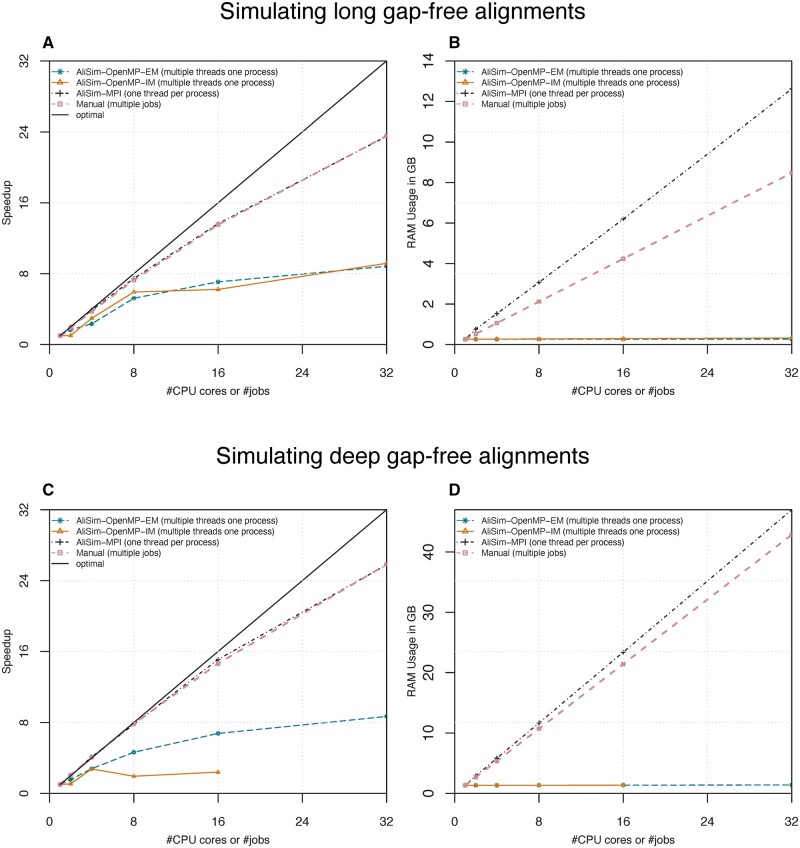
Strong scalings and peak RAM consumptions of the two AliSim-OpenMP algorithms, AliSim-MPI, and the manual approach (running multiple jobs) for long-alignment [30K sequences × 1M sites (A, B)]; and deep-alignment [1M sequences × 30K sites (C, D)] simulations without Indels. In deep-alignment simulations, AliSim-OpenMP-IM using 32 threads per process took an excessively long runtime; thus, we skipped that test to save the computational resources.

We first benchmark the pure multi-threading algorithms (AliSim-OpenMP) without multi-processing. Figure 5 shows the performance of the two AliSim-OpenMP algorithms using internal (IM) and external memory (EM). For long (gap-free) alignment simulations, when the number of threads is 8 or more, AliSim-OpenMP-IM obtained 5.9–to 9.2-fold speedups (compared with the sequential AliSim), while the speedups for AliSim-OpenMP-EM were slightly lower at 5.2–8.8 folds (Fig. 5A). In contrast, for deep (gap-free) alignment simulations, AliSim-OpenMP-EM obtained higher speedups (from 1.6 to 8.7 folds) than the IM variant (from 1.1 to 2.8 folds) (Fig. 5C). This is because the IM algorithm maintains an internal memory space shared between threads (Fig. 4) that may cause a bottleneck when there are many threads waiting to write to this shared memory, e.g. speedup for 8 and 16 threads is worse than four threads (Fig. 5C). Therefore, the two versions complemented each other.

Regarding the RAM consumption, our AliSim-OpenMP algorithms consumed approximately 0.3 GB and 1.4 GB RAM to simulate long and deep alignments, respectively (Fig. 5B and D). Due to a large number of nodes *N* in the phylogenetic tree, simulating deep alignments consumed more RAM than long alignments (see Section 2). Besides, with the default setting of λ (see Section 2), the number of threads insignificantly affected the memory consumption.

### 3.2 AliSim-MPI obtained high parallel efficiency

Next, we benchmark the pure multi-processing AliSim-MPI version, where each process is single-threaded. AliSim-MPI and the manual approach performed equally well, which achieved almost linear speedup with high parallel efficiency (Fig. 5A and C). With 32 CPU processes or jobs, they achieved roughly 24× and 26× speedups (73% and 81% parallel efficiencies) for long and deep-alignment simulations, respectively.

The RAM consumption grew, as expected, proportionally with the increasing number of processes (Fig. 5B and D). AliSim-MPI required 0.3–13 GB, and 1.3–47 GB RAM to simulate long and deep alignments, respectively, which were 0.2–4 GB more RAM than the manual approach.

### 3.3 AliSim-HPC achieved excellent strong scaling behavior

**Figure 6.**
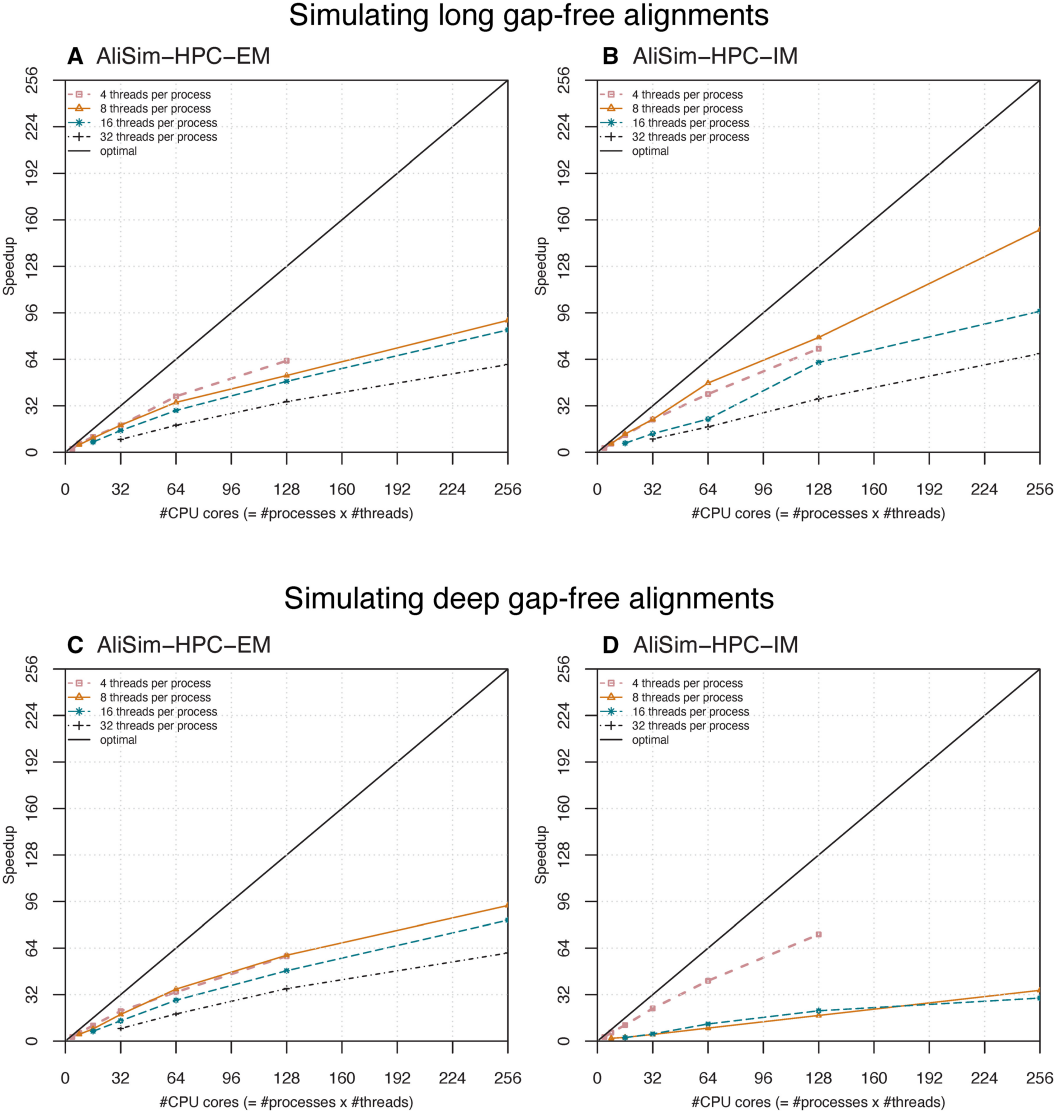
Strong scaling of AliSim-HPC-EM and AliSim-HPC-IM in long-alignment (A, B) and deep-alignment (C, D) simulations without Indels. In deep-alignment simulations, the curve of AliSim-HPC-IM using 32 threads per process is missing since these tests took excessively long runtime; thus, we skipped them to save the computational resources.

We now benchmark AliSim-HPC, which combines the benefits of AliSim-OpenMP (low RAM consumption) and AliSim-MPI (excellent speedups). Figure 6 illustrates the performance of the two variants AliSim-HPC-EM and AliSim-HPC-IM, using external and internal memory, respectively. For long-alignment simulations, while both variants achieved excellent strong scaling when increasing the total number of CPU cores (P*K), AliSim-HPC-IM (Fig. 6B) obtained higher speedups than AliSim-HPC-EM (Fig. 6A). For example, AliSim-HPC-IM reached 153-fold speedup using 32 processes × 8 threads (Fig. 6B), but AliSim-HPC-EM only reached 91-fold speedup. In fact, AliSim-HPC-EM achieved 182-fold speedup in step 1 (of Algorithm 2) compared with the sequential AliSim algorithm. However, AliSim-HPC-EM required an additional phase (step 2 in Algorithm 2) to concatenate temporary files, which took approximately the same amount of the runtime of step 1, thus reducing the overall performance of that algorithm.

For simulating deep alignments, AliSim-HPC-EM (Fig. 6C) often outperformed AliSim-HPC-IM (Fig. 6D). AliSim-HPC-EM obtained a 93-fold speedup compared with only 35-fold speedup of AliSim-HPC-IM for 32 processes × 8 threads. But interestingly, AliSim-HPC-IM with four threads per process performed better than the EM variant, obtaining 73-fold speedup for 32 processes × 4 threads compared with 58-fold speedup for AliSim-HPC-EM. Unfortunately, for this setting, we could not run our tests with a higher number of processes due to excessive memory requirements.

In summary, both versions of AliSim-HPC achieved excellent scaling behavior. The best setting of AliSim-HPC-EM and AliSim-HPC-IM reduced the wall-clock time from over 1 day to 11 and 21 min for simulating long and deep gap-free alignments, respectively.

### 3.4 The RAM consumption of AliSim-HPC increased with the number of processes

**Figure 7.**
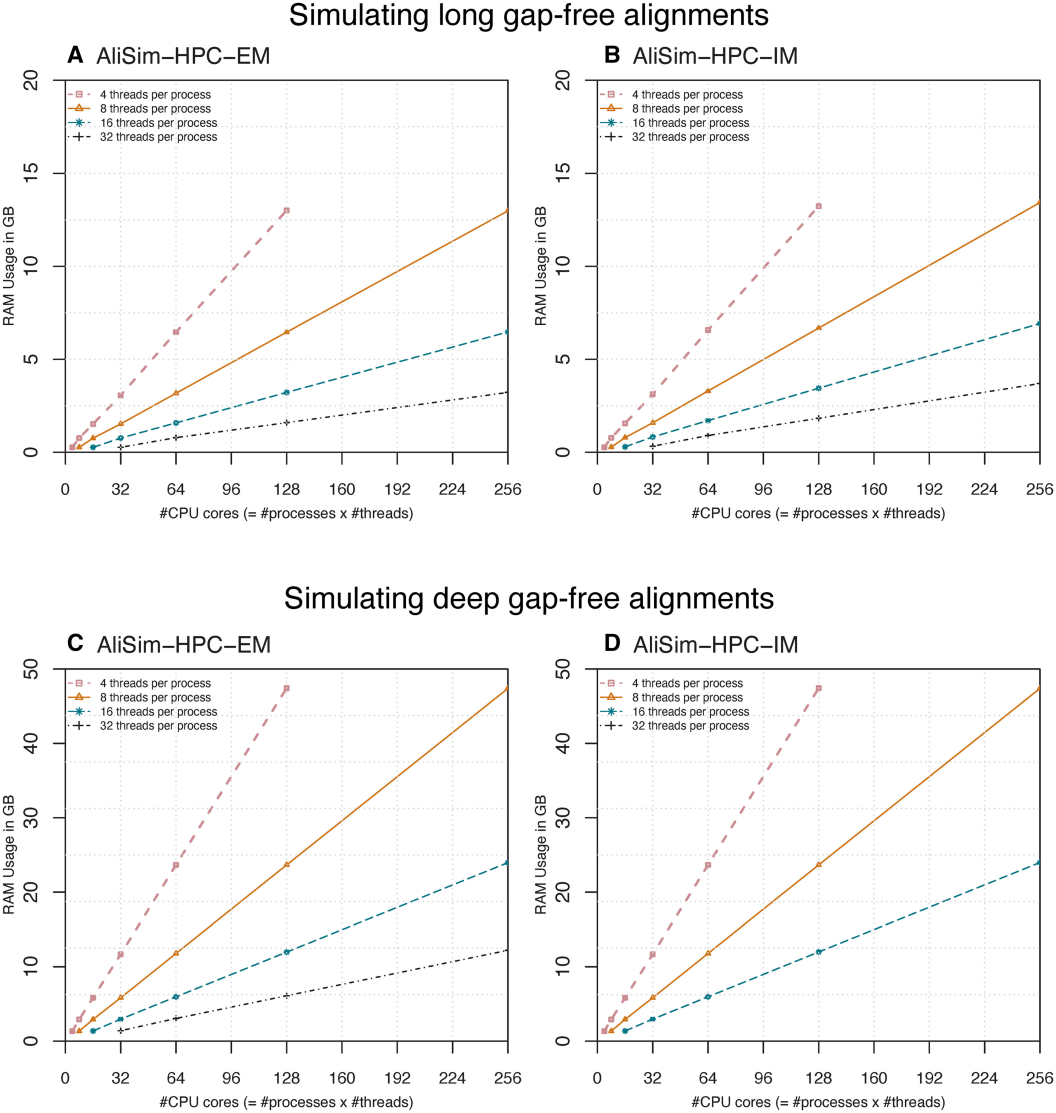
The memory footprint of AliSim-HPC-EM and AliSim-HPC-IM in long-alignment (A, B) and deep-alignment (C, D) simulations without Indels.

Figure 7 shows the memory footprint of AliSim-HPC-EM and AliSim-HPC-IM. As expected, the RAM consumption of the two variants increased with the number of processes *P*.

The memory footprints of the two variants of AliSim-HPC were almost identical. When increasing the number of processes from 1 to 32, they consumed 0.3–13.4 GB (Fig. 7A and B) and 1.3–47.4 GB RAM (Fig. 7C and D) to simulate long and deep alignments, respectively. With the same number of processes, the two variants of AliSim-HPC consumed the same amount of RAM as AliSim-MPI.

## 4 Discussion

This paper introduces AliSim-HPC, a high-performance sequence simulator for phylogenetics. We present two multi-threading algorithms to simulate a single large gap-free alignment with OpenMP, and an embarrassingly parallel scheme to simulate many alignments (with/without gaps) with MPI on a distributed-memory system. In the future, we would also like to extend AliSim-HPC to employ Single Instruction Multiple Data (SIMD) (Cardoso *et al.* 2017) and GPU-based parallelization.

With an appropriate setting of the number of threads per process, AliSim-HPC is highly efficient because it involves minimal inter-thread and no inter-process communications. Strong scaling experiments also showed that AliSim-HPC is scalable: we obtained 153-fold speedup when employing 256 CPU cores (32 MPI processes, each with eight threads) to simulate 100 large alignments without Indels; further speedup using more cores is achievable since it is not yet saturated (Fig. 6).

The pure MPI implementation allows users to simulate many MSAs within a single run, which is more convenient than the manual approach of launching several AliSim jobs. Whereas the pure OpenMP implementation allows us to save memory, which is useful for machines with limited RAM. AliSim-HPC combines both OpenMP with MPI, giving further advantage to optimize both runtimes and memory usage. To illustrate this, we ran the manual approach and different variants of AliSim-HPC on a machine with 32 cores available. Table 1 shows the runtimes and peak memory usage of these approaches when simulating 100 long alignments. The manual approach and AliSim-MPI are equally fast, but the manual approach is more memory-efficient, requiring 8.5 GB instead of 12.6 GB (the increase in RAM usage is purely due to the MPI library). AliSim-HPC offers many options to combine processes and threads per process. Among these options, AliSim-HPC using four processes with eight threads each is arguably the best option that only increases the runtime from 71 to 74 min while significantly reducing the RAM usage from 8.5 GB to 1.6 GB. AliSim-OpenMP (1 process × 32 threads) further reduces the memory usage to just 0.3 GB but with a trade-off of increasing the runtime to 182 min. It would be interesting to design a mechanism to automatically determine the best number of processes and threads to balance the trade-off between time and memory. That depends on many factors, including the size of the MSA (e.g. long versus deep) and the machine/cluster configuration, thus beyond the scope of our study.

**Table 1.** Runtimes and memory consumption of the manual approach and different AliSim-HPC settings when simulating 100 long (30K sequences × 1M sites) gap-free alignments on a computer with 32 cores available.

Approach	Settings	Runtime (min)	RAM (GB)
Manual	32 jobs	71	8.5
AliSim-MPI	32 processes × 1 thread	71	12.6
AliSim-HPC	16 processes × 2 threads	127	6.2
AliSim-HPC	8 processes × 4 threads	74	3.1
AliSim-HPC	4 processes × 8 threads	74	1.6
AliSim-HPC	2 processes × 16 threads	130	0.8
AliSim-OpenMP	1 process × 32 threads	182	0.3

The performance of the two AliSim-HPC variants greatly depends on the sequence length. Based on the experimental results, we recommend applying AliSim-HPC-IM for simulating long (gap-free) alignments. In contrast, to simulate short and moderate sequences (e.g. 30K sites) without gaps, AliSim-HPC-EM is preferable. However, determining whether an alignment is long or short is subjective and the performance of our algorithms also relies on the hardware (e.g. processor, SSD/HDD storage). Therefore, our future work also includes designing a mechanism to automatically select the best algorithm on the fly.

The OpenMP version has a limitation of not being able to simulate insertion–deletions. To alleviate this limitation, an alternative parallel strategy is to perform level order traversal on the tree so that sequences at same-depth nodes can be simulated simultaneously; each thread simulates a full-length sequence at a node. Besides, to simulate many alignments where the number of alignments is much greater than the number of CPU cores, we can also extend our AliSim-OpenMP algorithms so that each thread can simulate entire alignments independently to avoid the writing bottleneck.

Finally, the I/O operations are currently the bottleneck of our AliSim-OpenMP algorithms, which explains their far-from-perfect speedups (Fig. 5). Future work would employ parallel I/O techniques that will remove this bottleneck and make AliSim-HPC even more efficient for much larger-scale simulations.

## Data Availability

The data underlying this article are available in the Zenodo Repository at https://doi.org/10.5281/zenodo.7923875.

## References

[btad540-B1] Abadi S , Avram O, Rosset S et al ModelTeller: model selection for optimal phylogenetic reconstruction using machine learning. Mol Biol Evol 2020;37:3338–52. 32585030 10.1093/molbev/msaa154

[btad540-B2] Adell JC , Dopazo J. Monte Carlo simulation in phylogenies: an application to test the constancy of evolutionary rates. J Mol Evol 1994;38:305–9. 8006998 10.1007/BF00176093

[btad540-B3] Altekar G , Dwarkadas S, Huelsenbeck JP et al Parallel Metropolis coupled Markov chain Monte Carlo for Bayesian phylogenetic inference. Bioinformatics 2004;20:407–15. 14960467 10.1093/bioinformatics/btg427

[btad540-B4] Bouckaert R , Heled J, Kühnert D et al BEAST 2: a software platform for Bayesian evolutionary analysis. PLoS Comput Biol 2014;10:1–7. 10.1371/journal.pcbi.1003537 PMC3985171 24722319

[btad540-B5] Cardoso JMP , Coutinho JGF, Diniz PC. High-performance embedded computing. In: Cardoso JMP, Coutinho JGF, Diniz PC (eds), Embedded Computing for High Performance, Boston: Morgan Kaufmann, 2017, 17–56.

[btad540-B6] Cartwright RA. DNA assembly with gaps (Dawg): simulating sequence evolution. Bioinformatics 2005;21(Suppl 3):iii31–38. 16306390 10.1093/bioinformatics/bti1200

[btad540-B7] Chapman B , Gabriele J, van der pas R. Using OpenMP: Portable Shared Memory Parallel Programming (Scientific and Engineering Computation). Cambridge, Massachusetts: The MIT Press, 2007.

[btad540-B8] De Maio N , Boulton W, Weilguny L et al phastSim: efficient simulation of sequence evolution for pandemic-scale datasets. PLoS Comput Biol 2022;18:e1010056. 35486906 10.1371/journal.pcbi.1010056 PMC9094560

[btad540-B9] Felsenstein J. Inferring Phylogenies. Sunderland, MA: Sinauer Associates Inc, 2004.

[btad540-B10] Fletcher W , Yang Z. INDELible: a flexible simulator of biological sequence evolution. Mol Biol Evol 2009;26:1879–88. 19423664 10.1093/molbev/msp098 PMC2712615

[btad540-B11] Garland T , Dickerman AW, Janis CM et al Phylogenetic analysis of covariance by computer simulation. Syst. Biol 1993;42:265–92.

[btad540-B12] Goldman N. Statistical tests of models of DNA substitution. J Mol Evol 1993;36:182–98. 7679448 10.1007/BF00166252

[btad540-B13] Gropp W , Huss-Lederman S, Lumsdaine A et al MPI - The Complete Reference, The Mpi Ex Edition. Cambridge, Massachusetts: The MIT Press, 1998.

[btad540-B14] Gu X , Fu YX, Li WH et al Maximum likelihood estimation of the heterogeneity of substitution rate among nucleotide sites. Mol. Biol. Evol 1995;2:546–57. 10.1093/oxfordjournals.molbev.a040235 7659011

[btad540-B15] Harding EF. The probabilities of rooted tree-shapes generated by random bifurcation. Adv. Appl. Probab 1971;3:44–77.

[btad540-B16] Huelsenbeck JP. Performance of phylogenetic methods in simulation. Syst. Biol 1995;44:17–48.

[btad540-B17] Kozlov AM , Aberer AJ, Stamatakis A et al ExaML version 3: a tool for phylogenomic analyses on supercomputers. Bioinformatics 2015;31:2577–9. 25819675 10.1093/bioinformatics/btv184 PMC4514929

[btad540-B18] Kozlov AM , Darriba D, Flouri T et al RAxML-NG: a fast, scalable and user-friendly tool for maximum likelihood phylogenetic inference. Bioinformatics 2019;35:4453–5. 31070718 10.1093/bioinformatics/btz305 PMC6821337

[btad540-B19] Kuhner MK , Felsenstein J. A simulation comparison of phylogeny algorithms under equal and unequal evolutionary rates. Mol Biol Evol 1994;11:459–68. 8015439 10.1093/oxfordjournals.molbev.a040126

[btad540-B20] Legall N , Salvador LC. Selective sweep sites and SNP dense regions differentiate Mycobacterium bovis isolates across scales. Front Microbiol 2022;13:787856. 36160199 10.3389/fmicb.2022.787856 PMC9489834

[btad540-B21] Lemey P , Salemi M, Vandamme A-M (eds). The Phylogenetic Handbook: A Practical Approach to Phylogenetic Analysis and Hypothesis Testing. Cambridge: Cambridge University Press, 2009.

[btad540-B22] Leuchtenberger AF , Crotty SM, Drucks T et al Distinguishing Felsenstein zone from Farris zone using neural networks. Mol Biol Evol 2020;37:3632–41. 32637998 10.1093/molbev/msaa164 PMC7743852

[btad540-B23] Ling C , Cheng W, Zhang H et al Deep neighbor information learning from evolution trees for phylogenetic likelihood estimates. IEEE Access 2020;8:220692–702.

[btad540-B24] Ly-Trong N , Naser-Khdour S, Lanfear R et al AliSim: a fast and versatile phylogenetic sequence simulator for the genomic era. Mol. Biol. Evol 2022;39:msac092. 35511713 10.1093/molbev/msac092 PMC9113491

[btad540-B25] Mascagni M , Srinivasan A. Sprng: a scalable library for pseudorandom number generation. ACM Trans Math Softw 2000;26:436–61.

[btad540-B26] Minh BQ , Schmidt HA, Chernomor O et al IQ-TREE 2: new models and efficient methods for phylogenetic inference in the genomic era. Mol Biol Evol 2020;37:1530–4. 32011700 10.1093/molbev/msaa015 PMC7182206

[btad540-B27] Morel B , Kozlov AM, Stamatakis A et al ParGenes: a tool for massively parallel model selection and phylogenetic tree inference on thousands of genes. Bioinformatics 2019;35:1771–3. 30321303 10.1093/bioinformatics/bty839 PMC6513153

[btad540-B28] Nguyen L-T , Schmidt HA, von Haeseler A et al IQ-TREE: a fast and effective stochastic algorithm for estimating maximum-likelihood phylogenies. Mol Biol Evol 2015;32:268–74. 25371430 10.1093/molbev/msu300 PMC4271533

[btad540-B29] Rambaut A , Grassly NC. Seq-gen: an application for the Monte Carlo simulation of DNA sequence evolution along phylogenetic trees. Comput Appl Biosci 1997;13:235–8. 9183526 10.1093/bioinformatics/13.3.235

[btad540-B30] Schöniger M , von Haeseler A. Toward assigning helical regions in alignments of ribosomal RNA and testing the appropriateness of evolutionary models. J Mol Evol 1999;49:691–8. 10552050 10.1007/pl00006590

[btad540-B31] Smith ML , Hahn MW. Phylogenetic inference using generative adversarial networks. *Bioinformatics* 2022 10.1093/bioinformatics/btad543. PMC10500083 37669126

[btad540-B32] Suvorov A , Hochuli J, Schrider DR. Accurate inference of tree topologies from multiple sequence alignments using deep learning. Syst Biol 2020;69:221–33. 31504938 10.1093/sysbio/syz060 PMC8204903

[btad540-B33] Suvorov A , Schrider DR. Reliable estimation of tree branch lengths using deep neural networks. bioRxiv, 10.1101/2022.11.07.515518, 2022, preprint: not peer reviewed. PMC11326709 39102450

[btad540-B34] Tateno Y , Takezaki N, Nei M. Relative efficiencies of the maximum-likelihood, neighbor-joining, and maximum-parsimony methods when substitution rate varies with site. Mol. Biol. Evol 1994;11:261–77. 8170367 10.1093/oxfordjournals.molbev.a040108

[btad540-B35] Tavaré SMR. Some probabilistic and statistical problems in the analysis of DNA sequences. Lect Math Life Sci 1986;17:57–86.

[btad540-B36] Yule GU. A mathematical theory of evolution based on the conclusions of. Dr. J. C. Willis, F.R.S. Philos Trans R Soc Lond B 1925;213:21–87.

